# Metabolome and transcriptome related dataset for pheromone biosynthesis in an aggressive forest pest *Ips typographus*

**DOI:** 10.1016/j.dib.2022.107912

**Published:** 2022-02-08

**Authors:** Rajarajan Ramakrishnan, Amit Roy, Marco Kai, Aleš Svatoš, Anna Jirošová

**Affiliations:** a EXTEMIT-K, Faculty of Forestry and Wood Sciences, Czech University of Life Sciences Prague, Czech Republic; b Max Planck Institute of Chemical Ecology, Jena, Germany

**Keywords:** Pheromone biosynthesis, Bark beetle, Spruce, Gut tissue, De-novo, Omics

## Abstract

Eurasian spruce bark beetle, *Ips typographus*, is an aggressive pest among spruce vegetation. *I. typographus* host trees colonization is mediated by aggregation pheromone, consisting of 2-methyl-3-buten-2-ol and *cis*-verbenol produced in the beetle gut. Other biologically active compounds such as ipsdienol and verbenone have also been detected. 2-Methyl-3-buten-2-ol and ipsdienol are produced *de-novo* in the mevalonate pathway and *cis*-verbenol is oxidized from α-pinene sequestrated from the host. The pheromone production is presumably connected with further changes in the primary and secondary metabolisms in the beetle. To evaluate such possibilities, we obtained qualitative metabolomic data from the analysis of beetle guts in different life stages. We used Ultra-high-performance liquid chromatography-electrospray ionization-high resolution tandem mass spectrometry (UHPLC-ESI-HRMS/MS). The data were dereplicated using metabolomic software (XCMS, Camera, and Bio-Conductor) and approximately 3000 features were extracted. The metabolite was identified using GNPS databases and *de-novo* annotation in Sirius program followed by manual curation.

Further, we obtained differential gene expression (DGE) of RNA sequencing data for mevalonate pathway genes and CytochromeP450 (CyP450) genes from the gut tissue of the beetle to delineate their role on life stage-specific pheromone biosynthesis. CyP450 gene families were classified according to subclasses and given individual expression patterns as heat maps. Three mevalonate pathway genes and five CyP450 gene relative expressions were analyzed using quantitative real-time (qRT) PCR, from the gut tissue of different life stage male/female beetles, as extended knowledge of related research article (Ramakrishnan et al., 2022). This data provides essential information on pheromone biosynthesis at the molecular level and supports further research on pheromone biosynthesis and detoxification in conifer bark beetles.

## Specifications Table

**Table:** 

Subject	Omics: Metabolomics; Omics: Transcriptomics
Specific subject area	Molecular underpinning pheromone biosynthesis in *Ips typographus*.
Type of data	Table, Image, Chart.
How data were acquired	UHPLC-ESI-HR-MS/MS metabolomic analysis using MetaboAnalyst 5.0 RNA sequenced and analysed data using CLC workbench software Quantitative real-time PCR using the 2-ΔΔCt method
Data format	Analysed data.
Parameters for data collection	*Ips typographus* gut tissue. Different life stages.
Description of data collection	*Ips typographus* different life stages were collected from rearing and used for sample processing. Prepared samples were used for various downstream process such as RNA extraction for sequencing in the Ilumina platform and ethylacetate extraction for metabolomic analysis.
Data source location	Institution: Czech university of Life sciences City: Prague Country: Czech Republic and Institution: Max-Planck Institute for Chemical Ecology City: Jena Country: Germany
Data accessibility	*Ips typographus* different tissue UHPLC—HRMS/MS data: Dryad DOI: doi_10.5061_dryad.f7m0cfxws__v1 https://datadryad.org/stash/share/fw11pHlRK2WagKbXczgVb4wOqd2-gpMAATSOWD5EEEs *Ips typographus* different tissue RNA seq. data. accession number: PRJNA679450 https://www.ncbi.nlm.nih.gov/bioproject/PRJNA679450
Related Research article	R. Ramakrishnan, J. Hradecký, A. Roy, B. Kalinová, C. R. Mendezes, J. Synek, J. Bláha, A. Svatoš, A. Jirošová, Metabolomics and transcriptomics of pheromone biosynthesis in an aggressive forest pest *Ips typographus,* Insect Biochem. Mol. Biol. (2022)0965–1748. 10.1016/j.ibmb.2021.103680.

## Value of the Data

• Provided dataset of various metabolites and relative gene families from the gut tissue of *Ips typographus* is valuable for researchers with interest in studying different life stages of the bark beetle.
• Metabolomic data from UHPLC—HR-MS/MS analysis has provided insight of metabolites in different measurement modes and shared in dryad link. The acquisition methods of this data (using bioinformatics software programs such as GNPS, Sirus) are vital information for aiding similar analysis in the future and to developing bioinformatics tools for high-throughput metabolomics analysis.
• RNA seq. data revealed expression patterns of key gene families from the gut tissue of bark beetle life stages. This is valuable insight knowledge, allowing the researchers to follow up the present study with further research questions aligning with identified gene families.
• Information of standardized housekeeping genes and the quantitative real-time (qRT)- PCR data covers the knowledge gap, not included in the related research article.
• Henceforth, listed data in this article will be of added value for researchers to understand pheromone biosynthesis and metabolism of the related compounds in *I. typographus* and other bark beetle species and thus help to interrupt the beetle aggregation over spruce vegetation.

## 1 Data Description

The dataset we provided here is subjected to gut tissue of different life stages of the bark beetle, *I. typographus*. Ultra-high-performance liquid chromatography-electrospray ionization-high resolution tandem mass spectrometry (UHPLC-ESI-HR-MS/MS) data identified various metabolite compounds from the gut extracts using positive and negative ion mode and the results were shared in Table 1. Multivariate analysis of UHPLC—HR-MS/MS is shown in both positive and negative mode (Fig. 1a and b). Identified compounds clustering based on Partial least squares-discriminant analysis (PLS-DA) for different life stages of the beetle was given with different colours in Fig. 1a and b. Specific compounds masses responsible for the separation of life stages were listed with respective *m/z* ratio and retention time (RT) in Fig. 1a, B-G for positive mode, and in Fig. 1b, B-H for negative mode analysis. Fatty acids (C16 and C18) quantitative data over life stages are shown in Fig. 2. Proportions of identified metabolite classes from Table 1 are shown as Venn diagrams for both positive and negative ion mode in Fig. 3. Insight of di-glycosylated monoterpene alcohols was measured in both modes and masses were shared as peaks and CID spectra (Fig. 4). Mevalonate pathway compounds such as isopentyl-di-phosphate (IPP)/ dimethylallyl pyrophosphate (DMAPP) were visible in negative mode analysis with the help of synthetic standards and provided as peaks and CID spectra (Fig. 5).

**Table 1:** List of all identified metabolites from UHPLC-HR-MS/MS measurement in both positive and negative ion mode using data dereplication in GNPS and manually edited with the help of Sirius data annotations. Only possible precursor masses are listed in the table.

**Fig. 1a:**
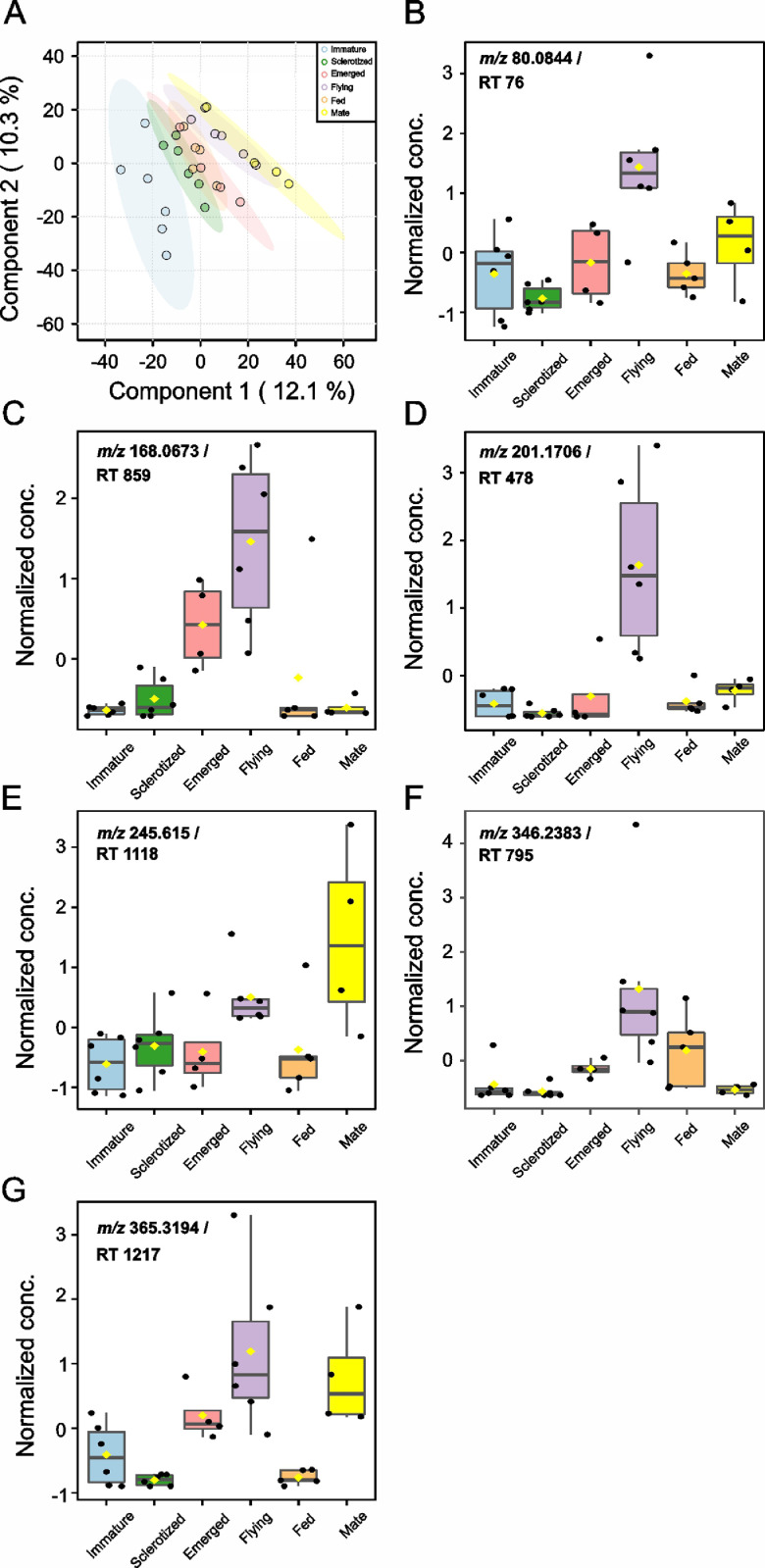
Uni- and multivariate analysis of UHPLC-ESI-HR-MS/MS analyzed metabolites extracted from different life stages of male *I. typographus. (*A) Partial least squares-discriminant analysis (PLS-DA) of *positive ion mode acquired data.* The colored areas represent 95% of the confidence interval between life stages of the beetle. (B–G) Correlation analysis of positive ion mode acquired mass features: (B) *m/z* 80.0844 at retention time (RT) 76 s, (C) *m/z* 168.0673 at RT 859 s, (D) *m/z* 201.1706 at RT 478 s, (E) *m/z* 245.615 at RT 1118 s, (F) *m/z* 346.2383 at RT 795 s, (G) *m/z* 365.3194 at RT 1217 s. PLS-DA and correlation analysis were performed using MetaboAnalyst 5.0 [8], an online tool for streamlined metabolomics data analysis.

**Fig. 1b:**
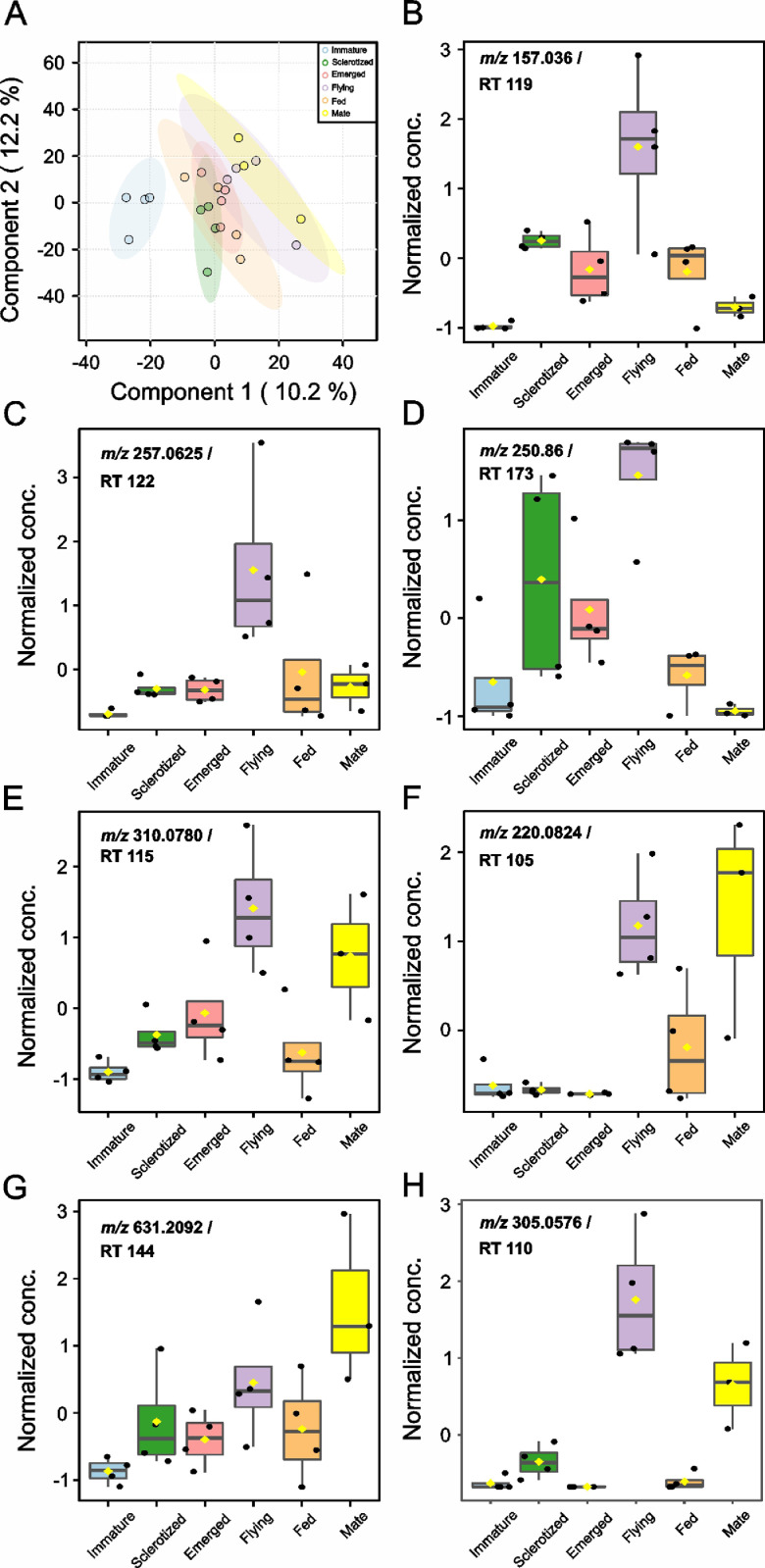
Uni- and multivariate analysis of UHPLC-ESI-HR-MS/MS analyzed metabolites extracted from different life stages of male *I. typographus. (*A) Partial least squares-discriminant analysis (PLS-DA) of *negative ion mode acquired data*. The coloured areas represent 95% of the confidence interval between life stages of the beetle (B–H). Correlation analysis of negative ion mode acquired mass features: (B) *m/z* 157.036 at retention time (RT) 119 s, (C) *m/z* 257.0625 at RT 122 s tentatively identified as 3-Dehydro-d-glucose 6-phosphate, (D) *m/z* 250.86 at RT 173 s, (E) *m/z* 310.0780 at RT 115 s tentatively identified as carboxy-neuraminic acid, (F) *m/z* 220.0824 at RT 105 s tentatively identified as N-Acetyl-d-Glucosamine, (G) *m/z* 631.2092 at RT 144 s. (H) *m/z* 305.0576 at RT 110 s. PLS-DA and correlation analysis were performed using MetaboAnalyst 5.0 [8], an online tool for streamlined metabolomics data analysis.

Furtherly, RNA sequencing data is shown as heatmaps for the expression pattern of the interested gene families between the life stages of the beetle. Primarily, insight of gene families such as the mevalonate pathway genes as heat map expression (Fig. 7) and further sesquiterpene compound producing genes from the pathway was described using the quantitative real-time (qRT)- PCR (Fig. 8). Identified 56 Cytochrome P450 genes (CyP450) and their overall expression given as a heat map (Fig. 9), with specific subclusters based on names acquired from Gene Ontology (GO) web reference using sequence similarity approach (Table 2). The expression pattern of the CyP450 gene seven subclusters was provided separately as heat map expression pattern in Figs. 10A, 10B, 10C and 10D which belongs to CyP450 6 like, CyP450 9e2 like, CyP450 9a1 like, CyP450 4 like and unknown CyP450 respectively. Furthermore, we studied qRT- PCR data of functionally known CyP450 gene known with sequence similarity from other bark beetle species and provided their expression level between mated male gut tissue and mated female gut tissues of *I. typographus* in Fig. 11. Added information of the housekeeping gene list with thirteen genes was ranked and provided after standardization (Fig. 6), which supports the related research article [5] for future gene study in mentioned tissue of the beetle.

**Fig. 2:**
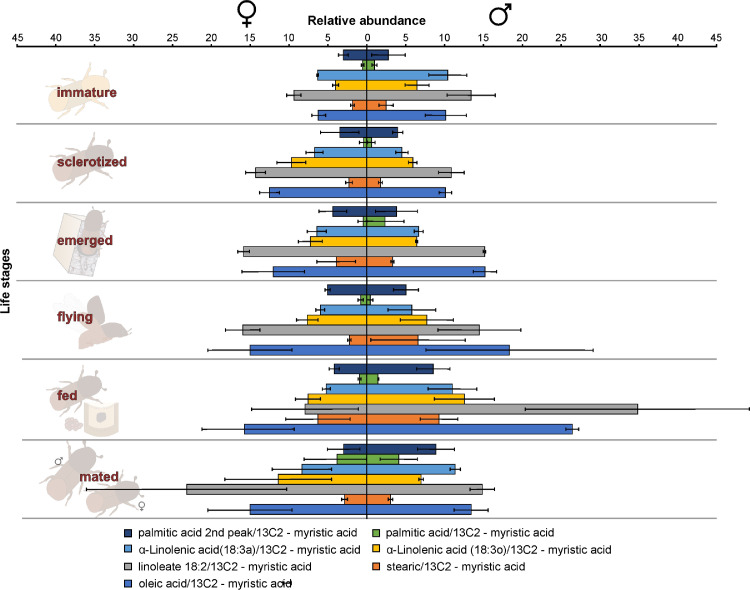
Intensities of five essential fatty acids normalized to ^13^C_2_-myristic internal standard signal intensities plotted over life stages of *I. typographus*. Guts extracts were analyzed on UHPLC-ESI-HR-MS/MS instrument in negative ion mode with TOP 5 scanning with one precursor ion scan and 5 MS/MS scans. Bars represent the standard error of the mean, *N* = 3.

**Fig. 3:**
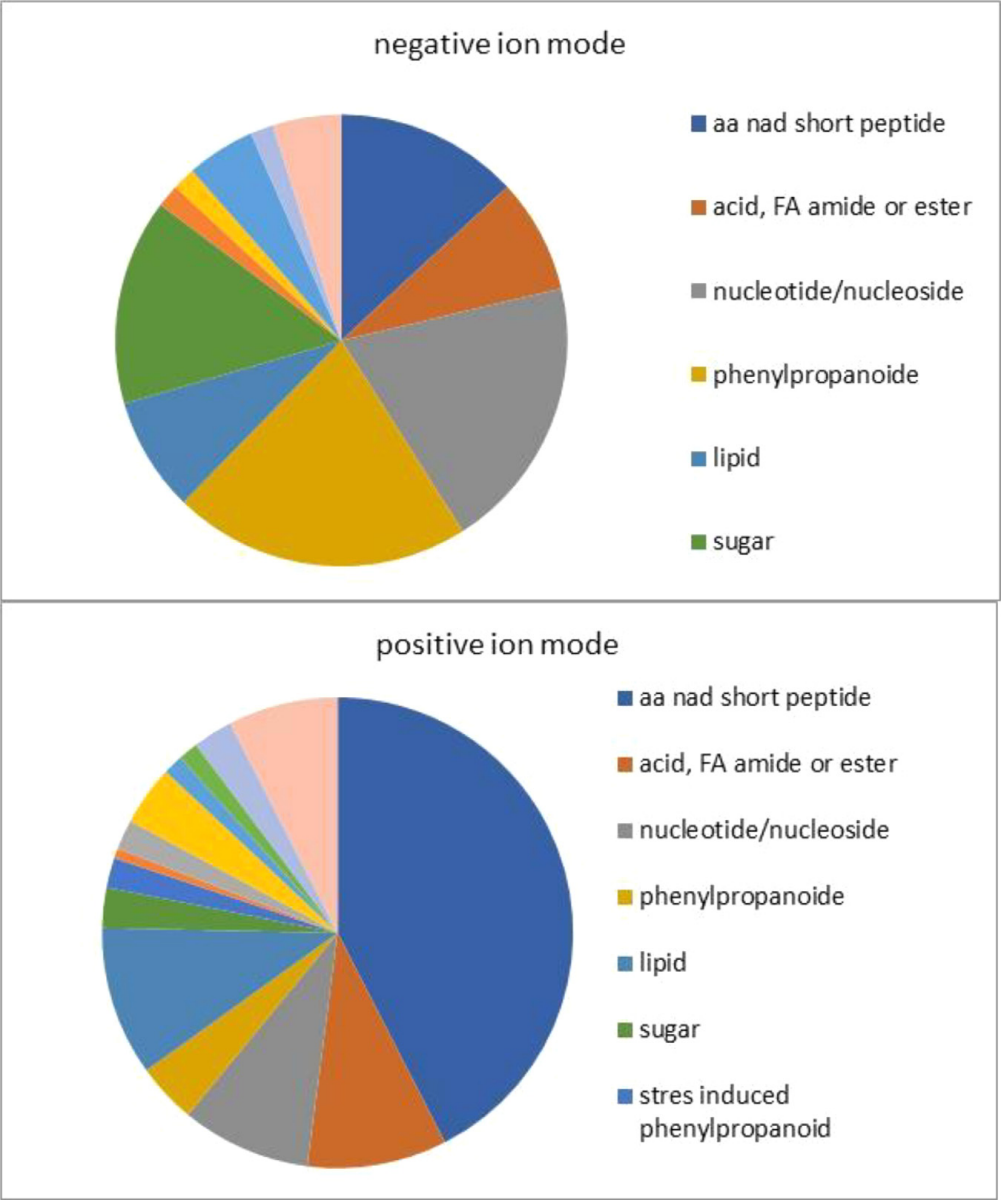
Venn diagram of metabolite classes from UHPLC-ESI-HR-MS/MS measurement in both positive and negative ion mode using data dereplication in GNPS and manually edited with help of Sirius data annotations. Complete annotation is available in Table 1.

**Fig. 4:**
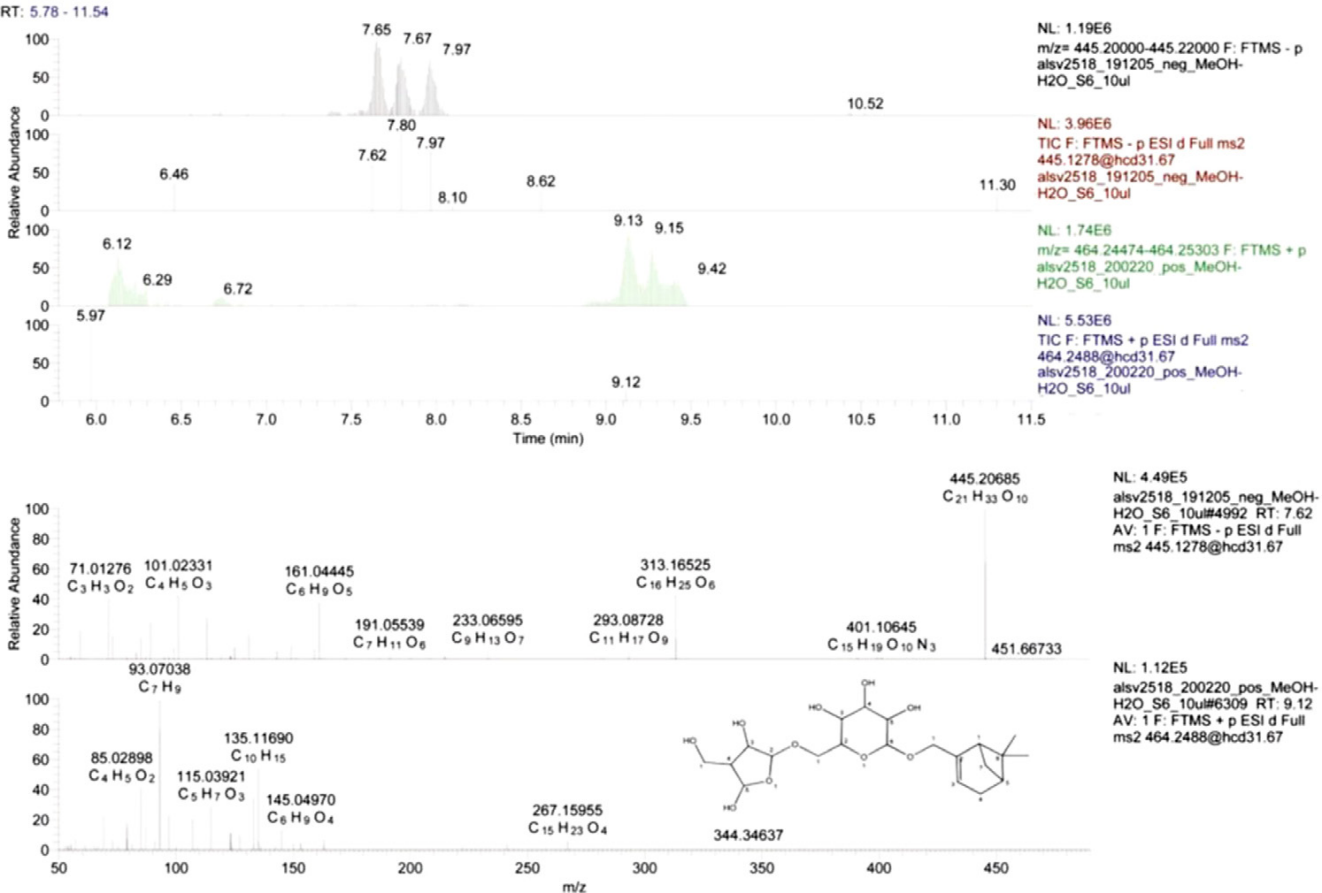
*A sections of UHPLC-ESI-HR-MS/MS traces plotted at specific mass ranges (upper panel)*: From top: negative ion mode, MS trace @ *m/z* 445.20–445.22 corresponding to [M-H]^−^ and MS/MS trace @ 445.127; positive ion mode MS trace @ *m/z* 464.24–464.25 corresponding to [*M*+NH_4_]^+^ and MS/MS trace @ 464.2488. Tree isobaric peaks are visible in both ion modes with similar intensities. The retention shift was due to a technical problem in one of the measurements. *CID spectra from isolated fixed precursor ion scans (lower panel)*: In negative ion mode, the intensive molecular peak is visible @ *m/z* 445.20685 with molecular composition C_21_H_33_O_10_. Ion @ *m/z* 161.0445 with molecular composition C_6_H_5_O_5_ is a deprotonated hexose. Two fragment ions C_16_H_25_O_6_ and C_11_H_17_O_9_ resulted from a loss of dehydro-pentose or monoterpene neutrals, respectively. In positive ion mode, molecular adduct peak is not visible, but low mass carbocation fragments (like C_10_H_15_ and C_7_H_9_) indicate monoterpene aglycone. The proposed structure of carbocation is given in the last CID spectra.

**Fig. 5:**
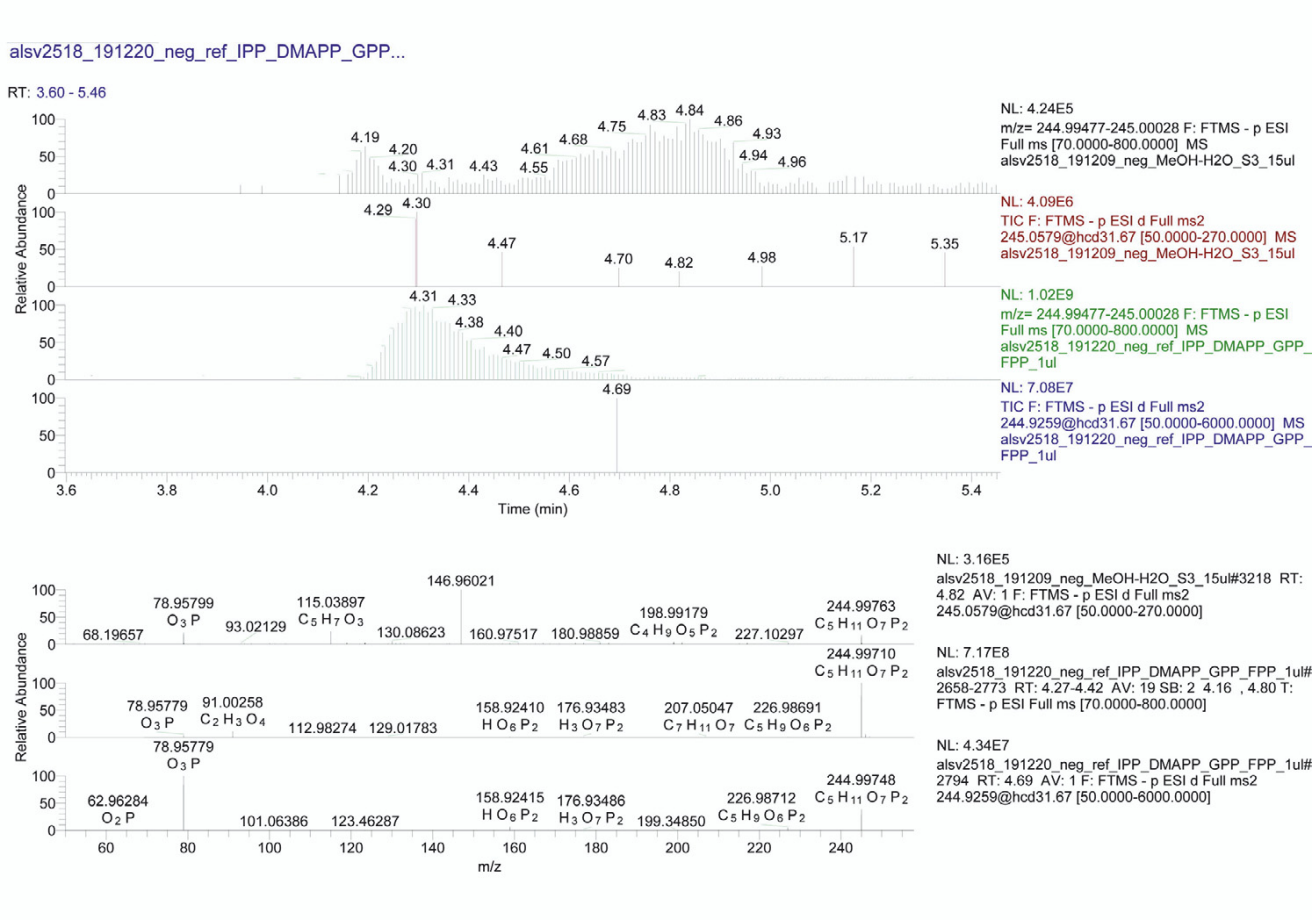
*A sections of UHPLC-ESI-HR-MS/MS traces plotted at specific mass ranges (upper panel)*: Comparison of the gut extract with a mixture of IPP and DMAPP synthetic standards measured under identical conditions. From top: negative ion mode, MS trace @ *m/z* 244.99–245.00 corresponding to [M-H]^−^ and MS/MS trace @ 245.06; mixture of standards, negative ion mode MS trace @ *m/z* 244.99–245.00 corresponding to [M-H]^−^ and MS/MS trace @ 244.93. Several peaks are visible in the expected retention window. Full scan MS spectra were collected from Rt 4.27–4.42 min. retention window. *CID spectra from isolated fixed precursor ion scans (lower panel)*: From top: acquired spectra at Rt 4.82 min showing *m/z* 244.99763 deprotonated molecular ion [M-H]^−^ and intense PO_3_^−^ ion at *m/z* 78.95799. The second plot is MS trace from Rt 4.27–4.42 min. showing intense deprotonated molecular ion [M-H]^−^ @ *m/z* 244.99710 with expected molecular composition. The plot below shows CID spectra of standards from isolated fixed precursor ion scans *m/z* 244.9259 showing a similar pattern as in the first plot from the gut extract.

**Table 2:** Identified 56 genes from CytochromeP450 gene family RNA seq data were clustered as shown in the table. Seven sub-cluster based on Multiple sequence alignment – Unipro UGENE v33.0 maximum likelihood is given as the table with the color difference. Sub-cluster 5, 6, and 7 were shown in similar colours since they were closely related. Cytochrome name replaced as Cy from RNA seq. data and the names were given based upon GO web reference using CLC workbench software. Tissue compared: fed male gut and immature male gut.

**Fig. 6:**
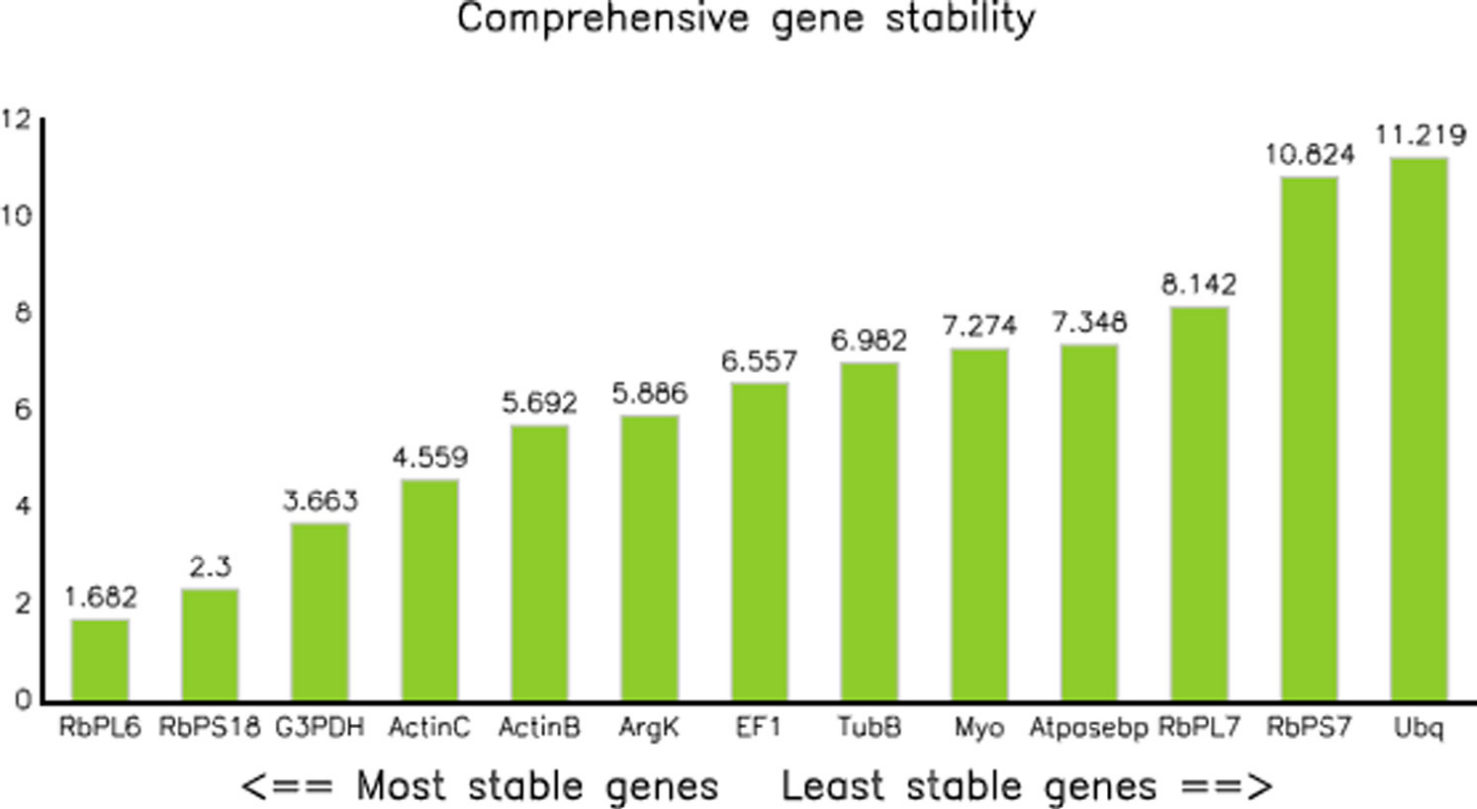
Validation of 13 housekeeping genes from *I. typographus* gut tissue using methods including bestkeeper, deltact, Genorm, and Normfinder. RbPL6- Ribosomal protein L6: RbPS18- Ribosomal protein S18; G3PDH-Glyceraldehydes 3-phosphate dehydrogenase; ActinC- actin-5C; ActinB- Actin; ArgK- Arginine Kinase; EF1- Elongation factor 1-alpha; TubB- tubulin beta chain; Myo- myosin heavy chain, non-muscle isoform X2; Atpasebp- V-type proton ATPase catalytic subunit A; RbPL7- Ribosomal protein L7; RbPS7- ribosomal protein S7; Ubq- ubiquitin-conjugating enzyme E2 J2. Most stable Ribosomal proteinL6 (RbPL6) and RibosomalproteinS18 (RbPS18) (The first two from left) were chosen for qRT -PCR data analysis. The values over the bar represent the normalization factor.

**Fig. 7:**
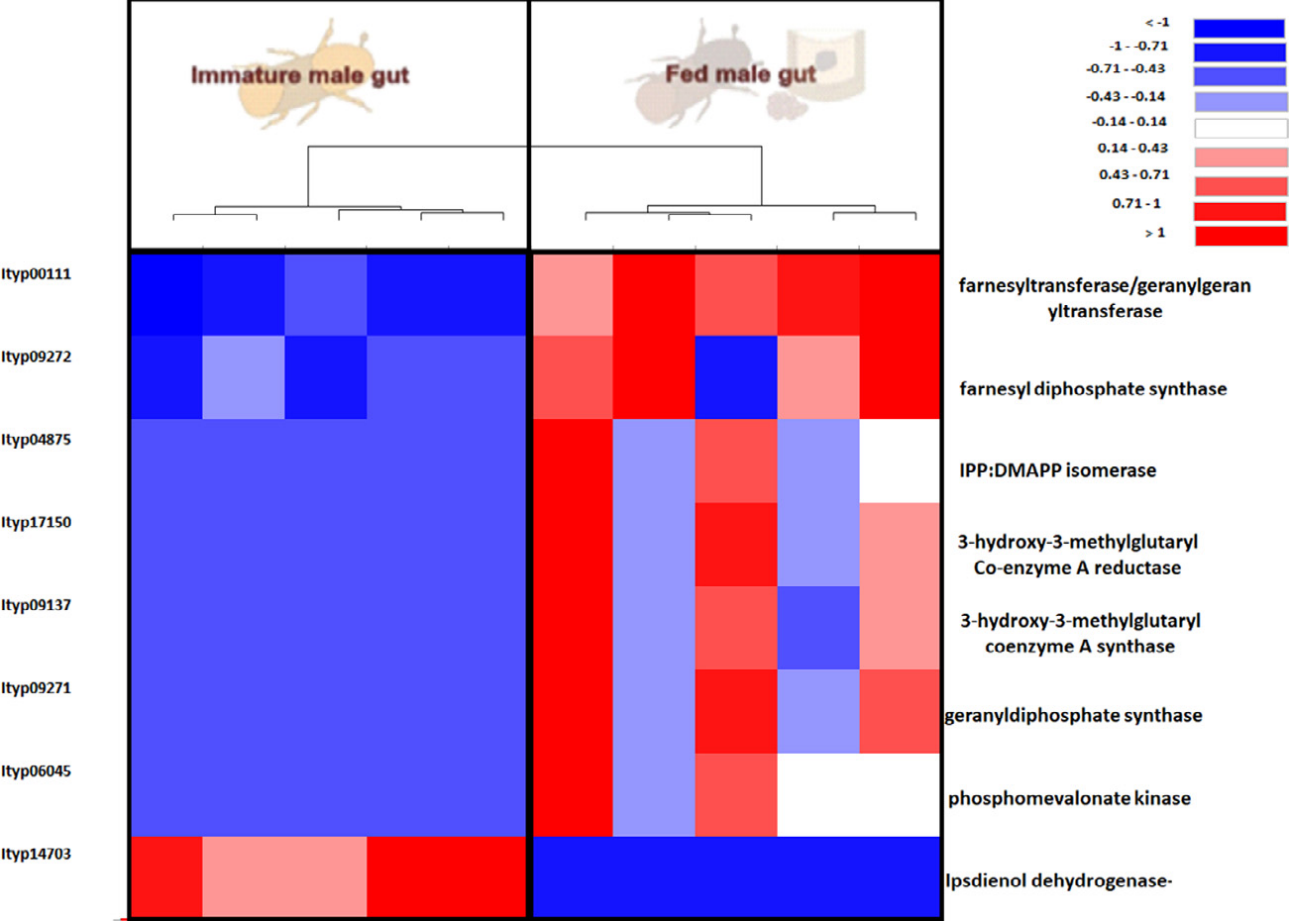
Heat maps representing eight mevalonate pathway gene expressions from RNA seq. data comparison. Tissue compared: fed male beetle gut vs immature beetle male gut. The tree cluster shows the tissue difference with 5 biological replicates. Red: high expression; Blue: low expression. Software used: CLC workbench and XLSTAT-Student 2020 Licensed version. Y-axis Left side was given with contig number of transcripts annotated with *I. typographus* genome. Y-axis Right side was labeled with possible gene name from Gene Ontology (GO) reference.

**Fig. 8:**
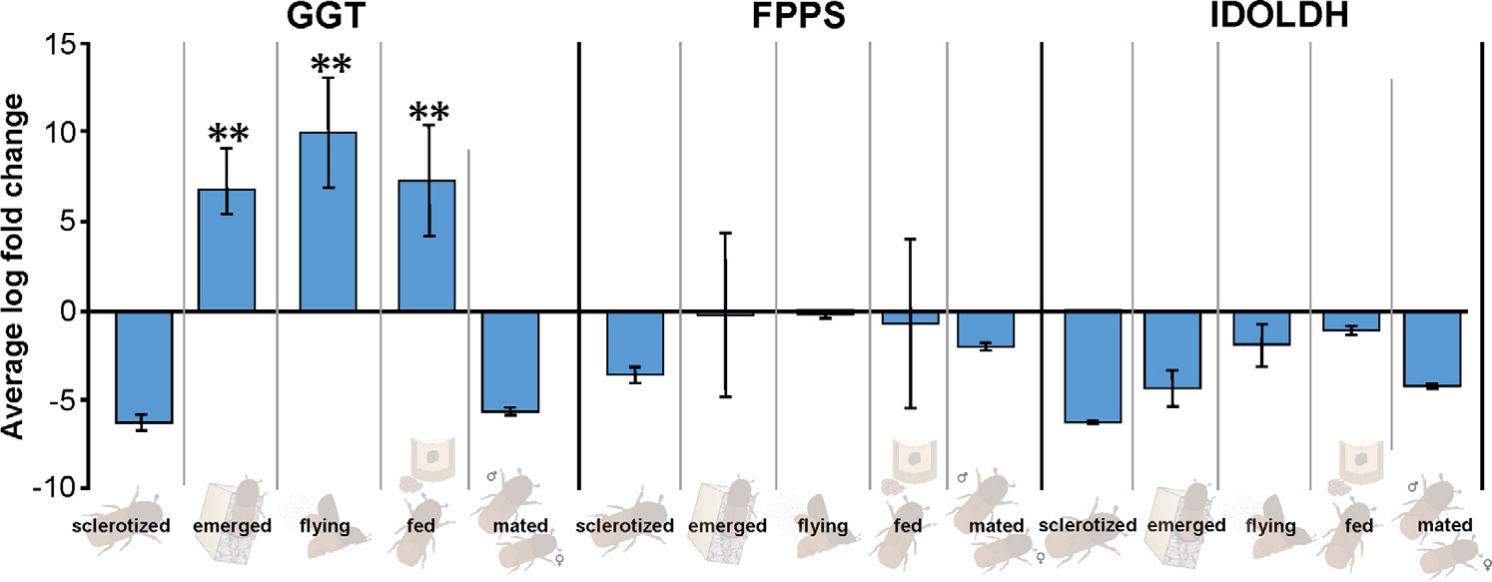
qRT-PCR data- different life stage male gut tissue analyzed for expression of three genes from mevalonate pathway: GGT- geranylgeranyl transferase, FPPS-Farnesyl diphosphate synthase, and IDOLDH- Ipsdienol dehydrogenase. These genes were chosen due to the least expressed in RNA-seq. data and involved in further sesquiterpene synthesis in the mevalonate pathway.

**Fig. 9:**
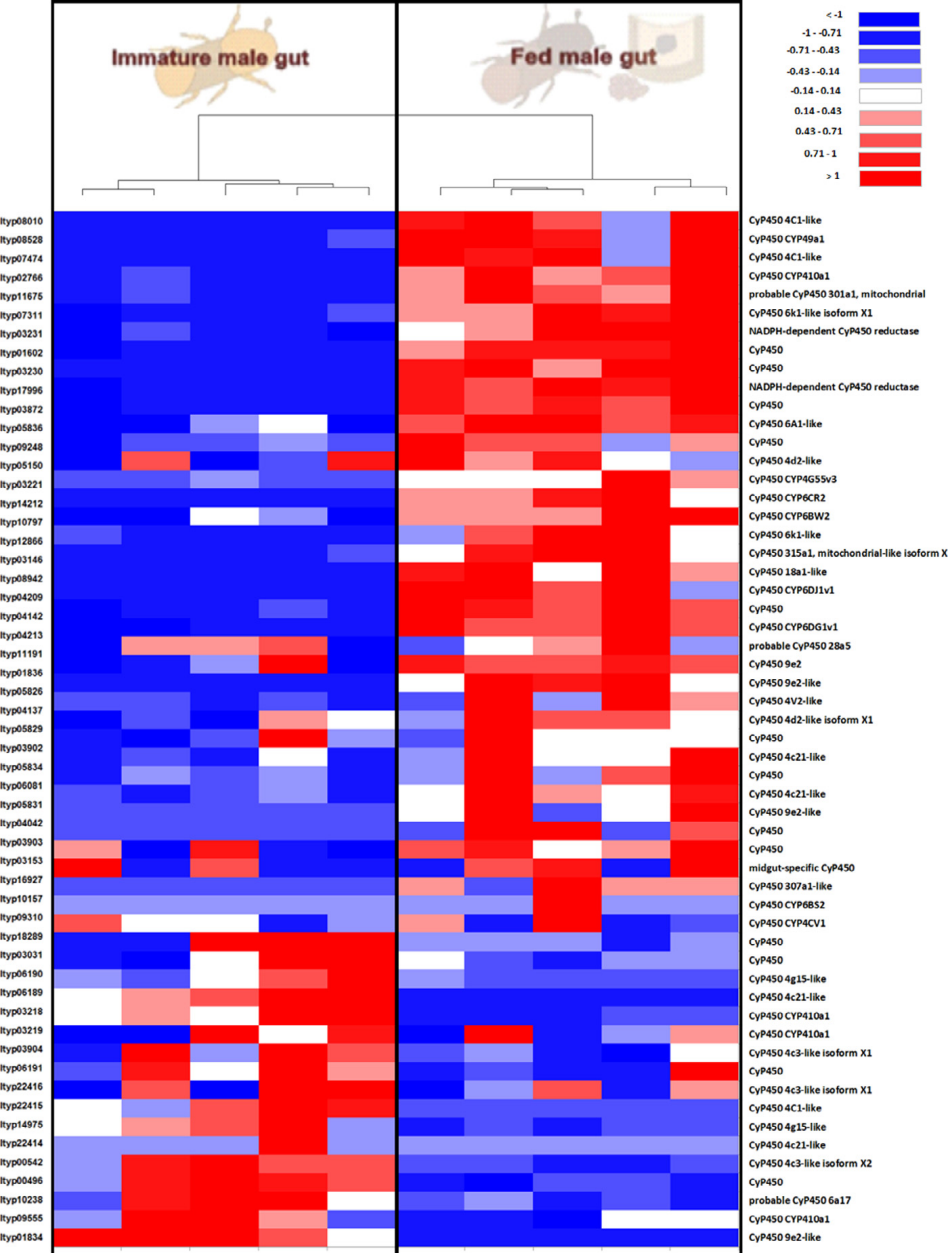
Heat map showing expression of identified Cytochrome P450 gene family with 56 genes from RNA seq. data. Tissue compared: fed male gut vs immature male gut. The tree clustered the tissue difference with 5 biological replicates. Red: high expression; Blue: low expression. Software used: CLC workbench and XLSTAT-Student 2020 Licensed version. Y-axis Left side was given with contig number of transcripts annotated with *I. typographus* genome. Y-axis Right side was labeled with a possible gene name from GO reference.

**Fig. 10A:**
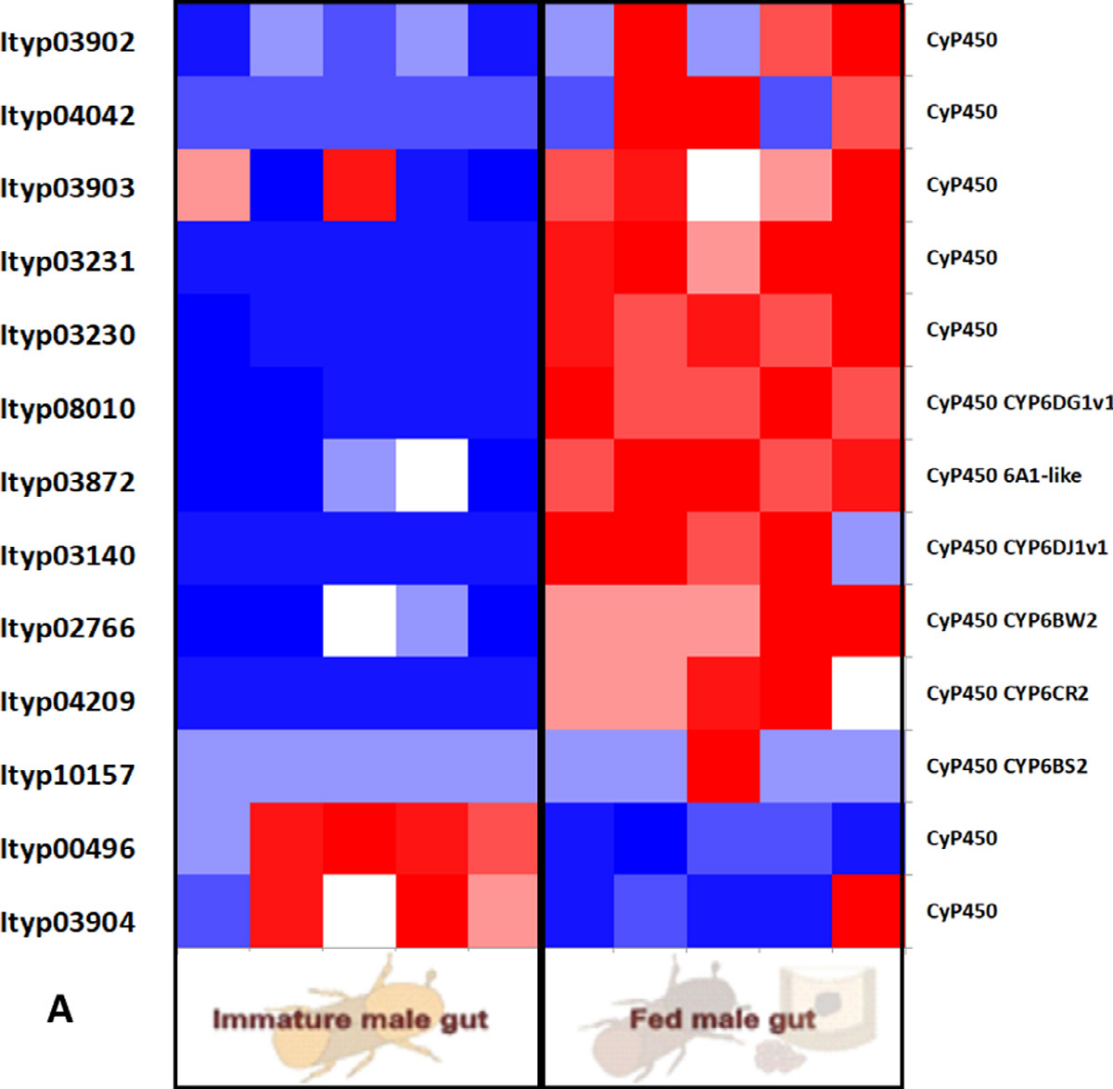
Heat map from RNA seq. data analysis showing the expression pattern of CyP450 gene_ sub-cluster 1 with possible 6 like gene family. Tissue compared: fed male gut vs immature male gut with 5 biological replicates. Red: high expression; Blue: low expression. Software used: CLC workbench and XLSTAT-Student 2020 Licensed version. Y-axis Left side was given with contig number of transcripts annotated with *I. typographus* genome. Y-axis Right side was labeled with a possible gene name from GO reference.

**Fig. 10B:**
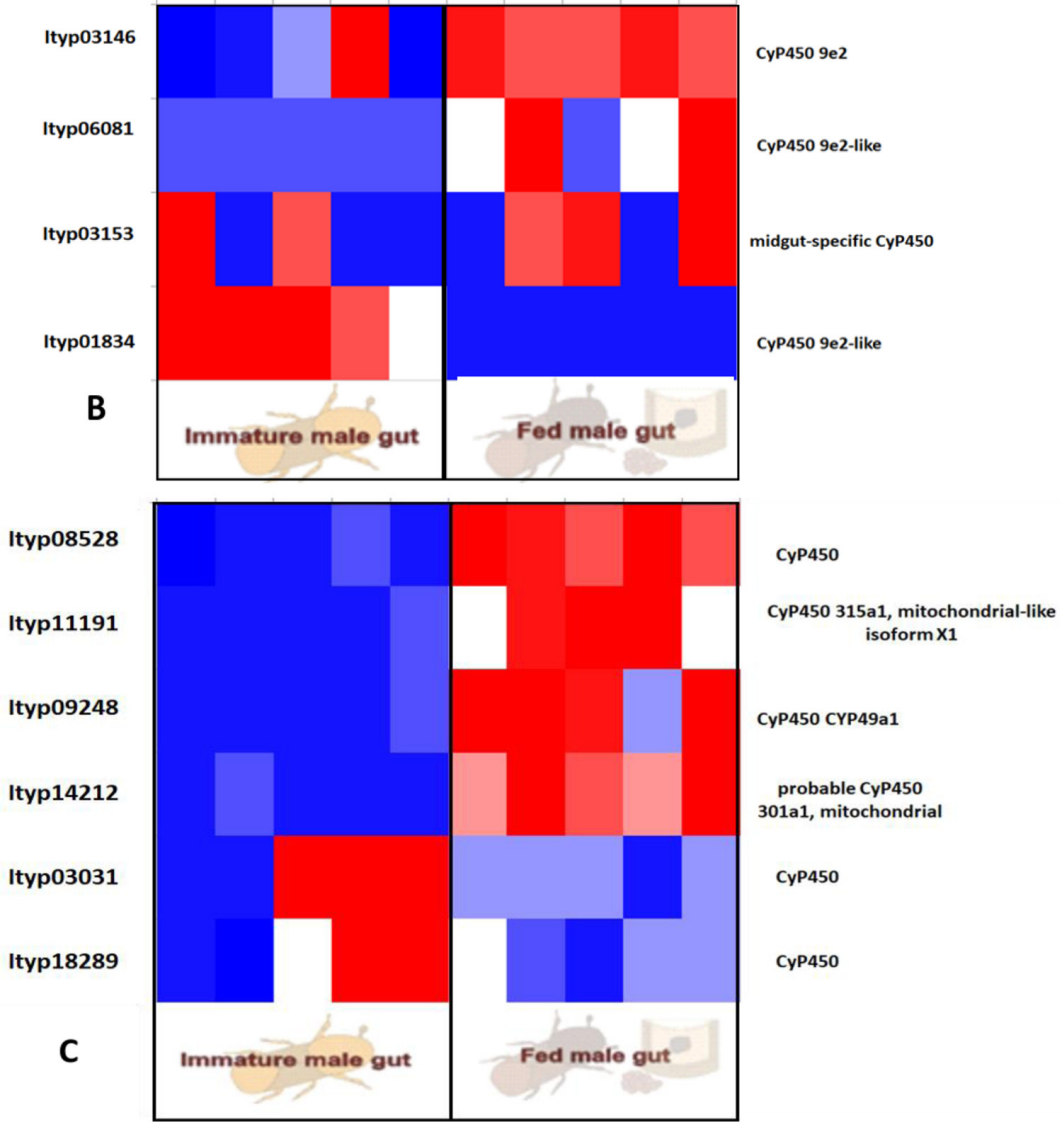
Heat map from RNA seq. data showing the expression pattern of CyP450 genes_ sub-cluster 2 with possible 9e2 like gene family. Fig. 10C: Heat map of TPM values showing CyP450 gene_ sub-cluster 3 with **possible 9a1** like gene family. Tissue compared: fed male gut vs immature male gut with 5 biological replicates. Red: high expression; Blue: low expression. Software used: CLC workbench and XLSTAT-Student 2020 Licensed version. Y-axis Left side was given with contig number of transcripts annotated with *I. typographus* genome. Y-axis Right side was labeled with the possible gene names from GO reference.

**Fig. 10C:**
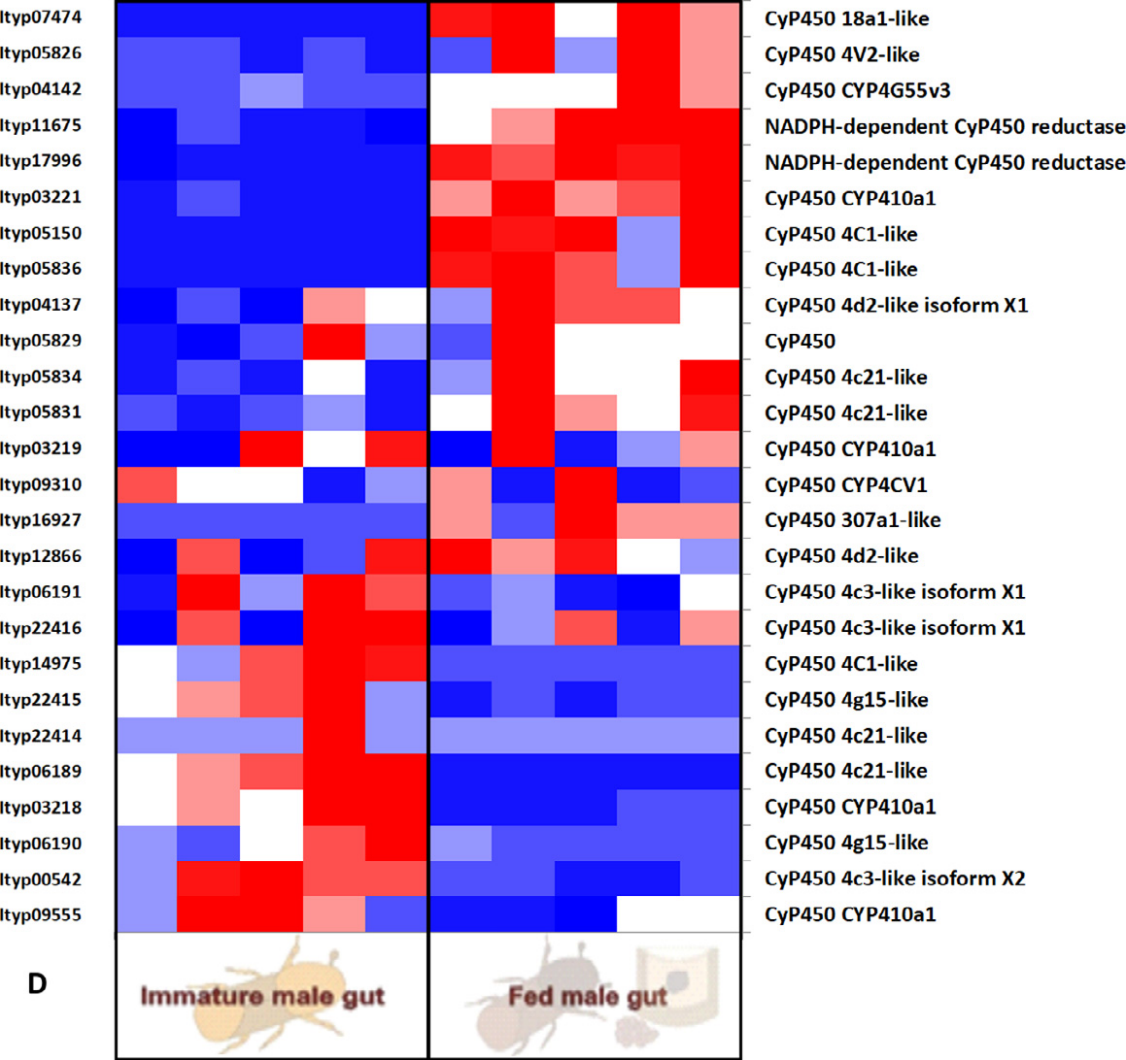
Heat map representing the expression pattern of *CyP450 gene_ sub-cluster 4* with 4 like gene family. Tissue compared: fed male gut vs immature male gut with 5 biological replicates. Red: high expression; Blue: low expression. Software used: CLC workbench and XLSTAT-Student 2020 Licensed version. Y-axis Left side was given with contig number of transcripts annotated with *I. typographus* genome. Y-axis Right side was labeled with the possible gene names from GO reference.

**Fig. 10D:**
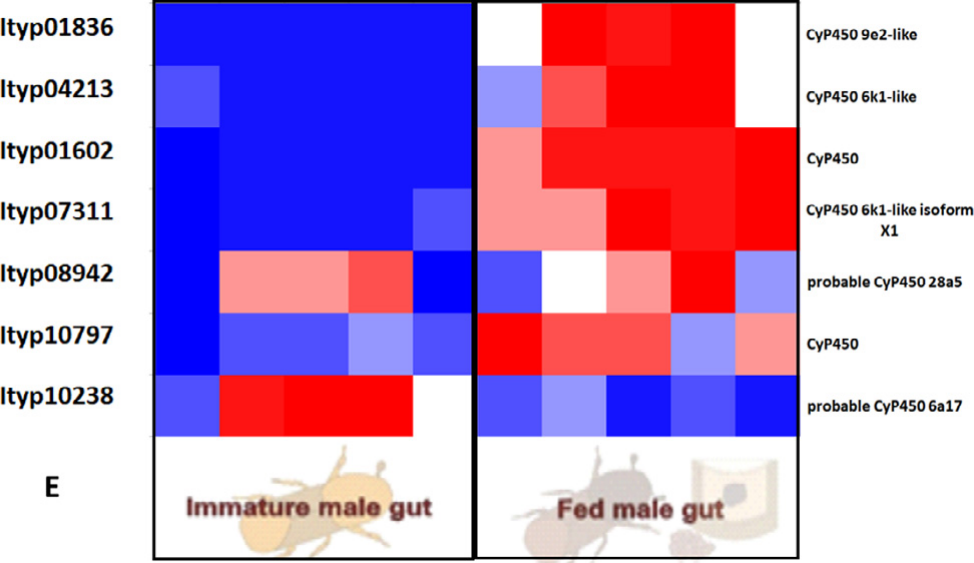
Heat map of TPM values representing *CyP450 gene_ sub-cluster 5, 6, and 7 with unknown CyP450* gene family. Tissue compared: fed male gut vs immature male gut with 5 biological replicates. Red: high expression; Blue: low expression. Software used: CLC workbench and XLSTAT-Student 2020 Licensed version. Y-axis Left side was given with contig number of transcripts annotated with *I. typographus* genome. Y-axis Right side was labeled with the possible gene names from GO reference.

**Fig. 11:**
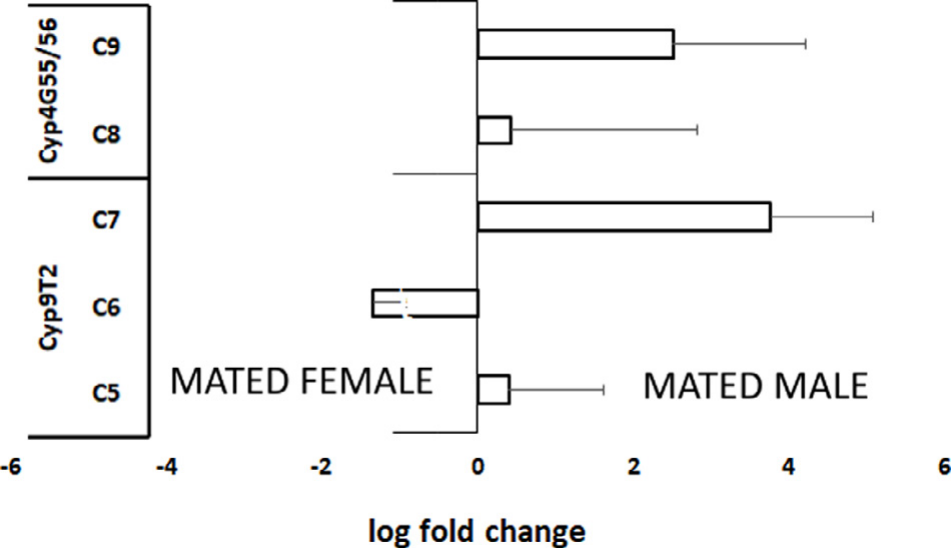
*qRT PCR data***:** Relative expression of Cyp450 genes C5-C9 in the mated male gut compared to mated female gut. C5-C7 are CyP450 gene candidates possibly involved in ipsdienol synthesis. C8-C9 are CyP450 gene candidates possibly involved in hydrocarbon synthesis. The number of biological replicates *N* = 4.

## 2 Experimental Design, Materials and Methods

Beetle rearing conditions and gut dissections were mentioned in relevant research article [5]. Before analysis, the guts were dissected from beetles of different life stages for further analysis.

### 2.1 Ultra-high-performance liquid chromatography- electrospray ionization -high resolution tandem mass spectrometry (UHPLC-ESI-HRMS/MS) analysis

Gut tissue was dissected (5 guts /sample) and collected in ethyl acetate (5 µl/gut) for storage at −80 °C before analysis. Gut extracts (solvent without gut) were removed for the nonpolar fraction. For polar extraction, rest of the solvent was removed by a gentle stream of nitrogen, and the remaining tissue was extracted (7 ml/gut) with MeOH/water/acetic acid (70/30/0.5 v/v) mixture containing ^13^C_2_-myristic acid (1 µg/ml) standard. After sonication on ice (5 min) the tissue was disrupted with a pre-chilled Eppendorf tip and sonicated for an additional 5 min. The samples were then centrifuged at 4000 RPM for 3 min and the supernatant was collected in a new vial with 100 µl glass insert. Gut extracts with nonpolar and polar fractions were used for UHPLC—HRMS/MS analysis [5].

UHPLC-ESI-HRMS/MS was performed at Ultimate 3000 series RSLC (Dionex) coupled with Q-Exactive HF-X mass spectrometer (Thermo Fisher Scientific, Waltham, USA). Water (solvent A) and acetonitrile (solvent B, LiChrosolv hyper grade for LC-MS; Merck, Darmstadt, Germany), both with 0.1% (v/v) formic acid (Eluent for LC-MS, Sigma Aldrich, Steinheim, Germany), were used for the binary solvent system. After injection of 10 µl extract, chromatographic separation was performed with a constant flow rate of 300 µl/min using an Acclaim C18 column (150 × 2.1 mm, 2.2 µm; Dionex, Borgenteich, Germany). Solvent gradients (B 0.5–100% v/v for 15 min; 100% B for 5 min; 100–0.5% v/v for 0.1 min; 0.5% for 5 min) were used. Ionization in HESI ion source was achieved by 4.2 kV cone voltage, 35 V capillary voltage, and 300 °C capillary temperature in the transfer tube in positive ion mode and 3.3 kV cone voltage, 35 V capillary voltage, and 320 °C capillary temperature in negative mode. Mass spectra were recorded in the positive and negative ion mode at *m/z* 80–800 mass range in duplicate. Date-dependent acquisition using TOP5 routine was used with one survey scan mass resolution 60,000 (HWFM), and 5 CID scans with 7500 resolution in ca 0.3 s. Colision-induced dissociation (hcd) of quadrupole selected precursor (0.8 Da mass window) was done in a collision cell at typically normalised fragmentation energy 30 eV. For identification pairs of the accurate mass of ions and their collision-induced ionization fragments with the retention time values were interpreted using software XCALIBUR (Thermo Fisher Scientific, Waltham, USA).

To identify metabolites, samples were compared and statistically evaluated using the software MetaboAnalyst 5.0 [3,8], and determined masses were compared with the database. The high-resolution LC-MS raw spectra were first centroided by converting them to mzXML format using the MS Convert feature of ProteoWizard 3.0.18324. Data processing was subsequently carried out with R Studio v1.1.463 using the Bioconductor XCMS package v 3.4.2 [1,^9^,10], which contains algorithms for peak detection, peak deconvolution, peak alignment, and gap filling. The resulting peak list was uploaded into MetaboAnalyst 5.0 [3,8], a web-based tool for metabolomics data processing, statistical analysis, and functional interpretation where statistical analysis and modeling were performed. Missing values were replaced using a (K-nearest neighbor) KNN missing value estimation. Data filtering was implemented by detecting and removing non-informative variables characterized by near-constant values throughout the experimental conditions by comparing their robust estimate interquartile ranges (IQR). Data was auto-scaled out of the 3020 mass features originally detected, using the Principal Least Square Discriminant Analysis PLS-DA [4].

To identify candidate metabolites, the individual mass features that contributed to the separation between the different classes were further characterized by applying a range of univariate and multivariate statistical tests to determine their importance including the PLS-DA importance variables, *t*-test, and Random Forest. This information, along with retention time, accurate mass, and MS/MS spectra were used to probe into existing literature and databases. MS/MS spectra files were also centroided and imported into GNPS [11] for spectral matches and classical molecular networking. The obtained database hits were manually evaluated. First, we looked for the quality of mass spectral peak matching, and later, we considered only reasonable hits. The hits related to contaminations were determined at this stage and are labeled in black. Obtained hits were collected in Table 1 and colored depending on the biosynthetic class of described compounds.

### 2.2 Differential gene expression (DGE) analysis

#### 2.2.1 RNA sequencing (RNA seq.) analysis

Dissected gut tissues were put in RNA*later* solution (10 µl/gut) and 10 guts per biological sample were used. RNA extraction was performed using the pre-optimized protocol [6,5]. The quality and quantity of the extracted RNA were evaluated using agarose gel and Qubit, respectively. Integrity was determined using the 2100 Bioanalyzer system (Agilent Technologies, Inc). Better quality RNA samples (RIN > 7) were sent for sequencing (150 bp paired-end reads, minimum 30 mil. reads per sample) to Novo-gene sequencing company, China [5].

Quantification of gene expression from the RNA sequence data was performed using CLC workbench was used to standardize by pre-optimized setting for mapping exon regions exclusively with genome reference. The biases in the sequences datasets and different transcript sizes were corrected using the TPM algorithm to obtain correct estimates for relative expression levels. Finally, Empirical analysis of differential Gene expression (DGE) was performed using the recommended parameters [6,7]. For DGE, FDR corrected p-value cut off < 0.05 and fold change cut off of ± 4 -fold as a threshold value for being significant. Differentially expressed genes were functionally annotated using the “cloud blast” feature within the “Blasto2GO plugin” in CLC Genomic Workbench. Nucleotide blast was done against the arthropod database with an E-value cut off 1.0E-10. Both, annex and GO slim was used to improve the GO term identification further by crossing the three GO categories (biological process, molecular function, and cellular component) to search for name similarities, GO term, and enzyme relationships within KEGG (Kyoto Encyclopedia of Genes and Genomes) pathway database [5].

#### 2.2.2 Quantitative real-time-PCR (qRT-PCR) analysis

qRT-PCR was used to validate the list of selected genes. Primers were designed using IDT's primer design software as given in Tables 3 and 4. cDNA for RT-qPCR was synthesized using RNA from respective gut tissue samples. cDNA was synthesized using an M-MLV reverse transcriptase kit following the manufacturer protocol. Resulted in cDNA samples were diluted up to 1:4 with nuclease-free water, and qRT-PCR was performed using SYBR™ Green PCR master mix (Applied Biosystems, USA) under the following parameters: 95 °C for 3 min, 40 cycles of 95 °C for 3 s, 60 °C for 34 s [2,^7^,5]. Melt curves were generated to ensure single product amplification. The expression levels of the target genes were calculated using the 2-ΔΔCt method with optimized two housekeeping genes as a reference for normalization with four biological replications.

**Table 3:** Primers designed for mevalonate pathway gene family in IDT primer quest designing tool with primer length of 18–25 bp Tm-55–65, GC-50–60%, Amplicon size:100–150 bp. Modified from Table S2 of relative research article [5].

S.no.	Contig numbers.	Gene names	Primer sequence	Length	Tm	GC%	Amplicon
1	Ityp00111	GGT-F	GGAACACCCAGTTGTCTCTA	20	60.932	50	
		GGT-R	GACTGGCTGCTGTCTTTG	18	60.369	55.556	128
2	Ityp09272	FPPS- F	GGGAACGGACATTCAAGAC	19	60.183	52.632	
		FPPS- R	GTTCTGACCTGCCGTAATG	19	60.199	52.632	110
3	Ityp14703	IDLDH -F	ATCCTCTCCTTGACCTATCC	20	59.8	50	
		IDLDH -R	ATCGGAGTGTCGCAGATA	18	59.862	50	92

**Table 4:** Primers designed for selected nine CyP450 gene families (C1-C9). IDT primer quest designing tool was used with primer length of 18–25 bp Tm-55–65, GC-50–60%, Amplicon size:100–150 bp. Modified from Table S3 from relative research article [5].

Label	Primer names with contig numbers.	Primer sequence	Length	Tm	GC%	Amplicon
C5	qcyp_1834-F	CCTTTCCTTGATCGACTCTG	20	59.722	50	
	qcyp_1834-R	CCCTGTGGAACGGATAAAC	19	59.951	52.632	121
C6	qcyp_3146-F	GAAAGTGGCCTCCTGTTG	18	60.111	55.556	
	qcyp_3146-R	CATGTCGCCCACGTTAAG	18	60.393	55.556	107
C7	qcyp_3153-F	GTGAGCGTTGGAAGGAAA	18	59.858	50	
	qcyp_3153-R	CACTTCTGTTGGTCCGTTAG	20	60.152	50	140
C8	qcyp_4140-F	CTGAAGTGCCCGAAGAAC	18	60.047	55.556	
	qcyp_4140-R	CATCAACATCCAGGTCATCC	20	60.179	50	123
C9	qcyp_4142-F	AACCGCAATGGGTGTAAG	18	59.999	50	
	qcyp_4142-R	GAGGATGTCTGGATAGAGGTAG	22	60.322	50	127

## Ethics Statement

We have performed all beetle experiments comply with the ARRIVE guidelines and are being carried out in accordance with the U.K. Animals (Scientific Procedures) Act, 1986 and associated guidelines, EU Directive 2010/63/EU for animal experiments, or the National Institutes of Health guide for the care and use of laboratory animals (NIH Publications No. 8023, revised 1978).

## CRediT authorship contribution statement

**Rajarajan Ramakrishnan:** Formal analysis, Writing – original draft. **Amit Roy:** Formal analysis, Writing – review & editing. **Marco Kai:** Formal analysis. **Aleš Svatoš:** Formal analysis, Writing – review & editing. **Anna Jirošová:** Formal analysis, Supervision, Writing – review & editing.

## Declaration of Competing Interest

None.
